# A rare subtype of the extensor digitorum brevis manus muscle: description and clinical relevance

**DOI:** 10.1007/s00276-026-03947-2

**Published:** 2026-07-15

**Authors:** Mateus Lacerda de Souza, Josemberg da Silva Baptista

**Affiliations:** Laboratory of Applied Morphology (LEMA), Department of Morphology, Universidade Federal do Espírito Santo, Marechal Campos Avenue, 1468, Maruipe, Vitoria, Espirito Santo 29043-900 Brazil

**Keywords:** Extensor digitorum brevis manus, anatomical variation, hand anatomy, extensor muscles, surgical anatomy

## Abstract

**Objective:**

The extensor digitorum brevis manus (EDBM) is an infrequent anatomical variation on the dorsum of the hand, with an estimated prevalence of 2%. Morphologically, type III is the rarest variant. The purpose of this study is to provide a detailed morphological description of a rare specimen of the EDBM, classified as type III, and to analyze its biomechanical implications and clinical relevance in the differential diagnosis.

**Materials and methods:**

A meticulous anatomical dissection was performed on the dorsal surface of the left hand of a male cadaver. Morphometric analysis was conducted using a digital caliper to record the precise dimensions of the muscle belly and its associated tendon.

**Results:**

An accessory muscular structure was identified deep to the tendons of the extensor digitorum muscle, located medially to the extensor indicis muscle. The muscle originated from the dorsal radiocarpal ligament at the distal portion of the radius and inserted into the medial margin of the extensor expansion of the middle finger. This configuration corresponds to the type III variant by Ogura et al., which represents the rarest variant described in the literature.

**Conclusions:**

The EDBM is a significant anatomical variation often misdiagnosed as a ganglion or synovial cyst due to lack of awareness among clinicians. Understanding its rarest subtypes, such as type III, is essential for improving clinical practices and surgical management thereby enhancing the quality of life for symptomatic patients.

## Introduction

The extensor digitorum brevis manus muscle (EDBM) is an infrequent anatomical variation located on the dorsum of the hand. Although the initial term applied for this muscle was extensor brevis digiti indicis vel medii, since 1875 the term EDBM has become most commonly accepted. In systematic reviews that analyzed cadaver studies, its prevalence was estimated between 1.96% and 2.3% [1–3]. Morphologically, the EDBM has subtypes categorized by the location of its distal insertion and by its coexistence or non-coexistence with the extensor indicis proprius (EIP). Following the classification established by Ogura et al. in 1987, this variation is described into three groups: type I is characterized by the insertion of the EDBM in the extensor hood of the index finger in association with the absence of the EIP; type II manifests when both muscles are present and insert into the second finger, further subdivided into subtypes IIa, IIb, and IIc; finally, type III, which represents the rarest configuration, is defined by the presence of the EIP while the EDBM shifts its distal insertion to the extensor hood of the middle finger [4].

Clinically, the main symptom reported is chronic pain on the dorsal aspect of the wrist [5]. The literature documents recurrent errors in diagnosis and treatment because of the lack of knowledge about this variant [6]. Once the presence of the anatomical variation with symptomatic presentation is confirmed, surgical interventions demonstrate effective reversibility [7].

## Objective

This study aims to describe a specimen with EDBM as well as to analyze and discuss the clinical manifestations and biomechanical implications inherent to this variation, aiming to contribute to the improvement of clinical practices and the effectiveness of management in symptomatic patients with this muscle.

## Materials and methods

**Fig. 1:**
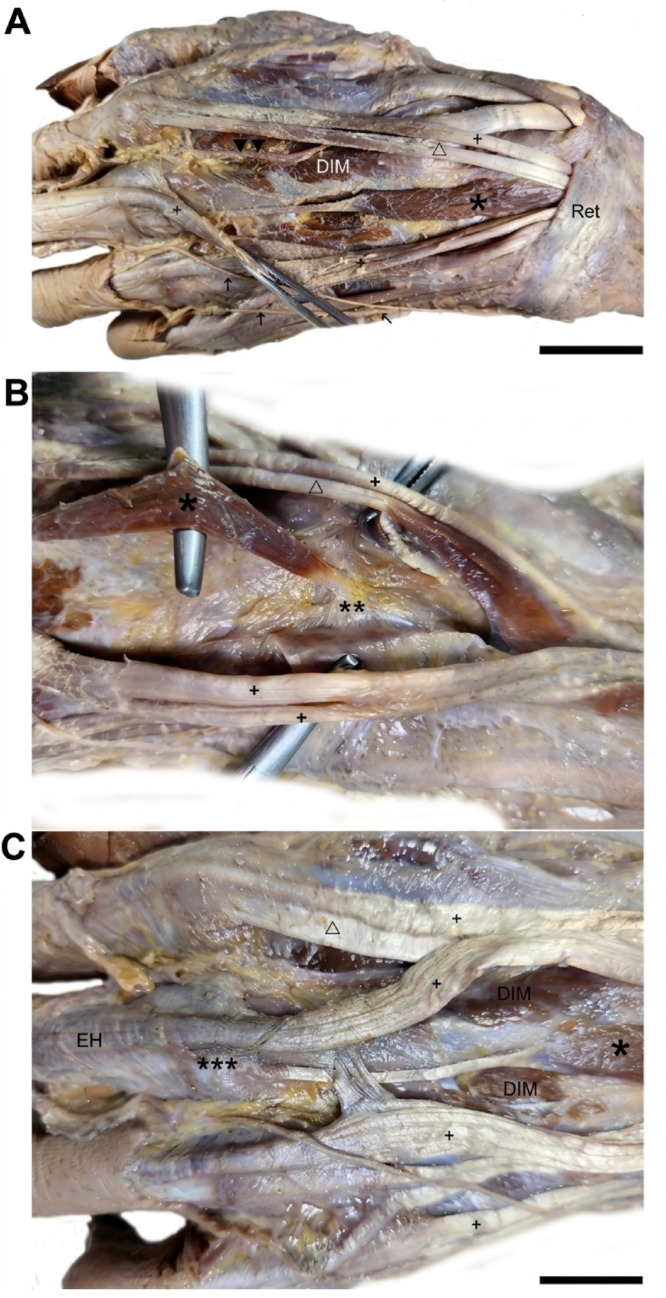
Photographs of the dorsum of the left hand. Ret.: extensor retinaculum; *: extensor digitorum brevis manus muscle; +: tendons of the extensor digitorum muscle; Triangle: extensor indicis proprius muscle; DIM: dorsal interosseous muscles; Black arrow: dorsal branch of the ulnar nerve; **: proximal attachment of the extensor digitorum brevis manus muscle; ***: distal attachment of the extensor digitorum brevis manus muscle; EH: extensor hood. Scale bars: A = 2 cm; B and C = 1 cm

During a dissection session at the gross anatomy course, the presence of an accessory muscular structure located on the dorsal surface of the left hand was evidenced. This hand belonged to a male cadaver, estimated to be between 60 and 70 years old (Fig. 1), previously fixated in a 4% formalin solution. This specimen is part of the collection of the Morphology Department of the Universidade Federal do Espirito Santo and was donated by the Coroner’s Department of Espirito Santo through a Brazilian law (8.501/1992). A meticulous dissection methodology of the region was performed to describe the findings. The aforementioned manifestation presented a strictly unilateral character. The morphometric analysis was performed using a caliper (King Tool, China).

## Results

During the anatomical dissection and morphological analysis of the dorsum of the hand, the tendons of the extensor pollicis longus, extensor carpi radialis longus, extensor carpi radialis brevis, extensor digitorum, extensor indicis, and extensor digiti minimi muscles were identified. Subsequently, the dorsal interosseous muscles, the dorsal branch of the ulnar nerve, the extensor retinaculum, and the extensor hood apparatus were observed, as observed during dissection (Fig. 1A).

After removing the tendon of the extensor digitorum muscle, specifically the bundle responsible for extensor expansion of the middle finger, the EDBM was identified, located medially to the tendon of the extensor indicis muscle and deep to the tendon of the extensor digitorum muscle, at the level of the third finger (Fig. 1B).

The muscular belly of the EDBM was superimposed on the dorsal interosseous muscles, positioned between the medial and lateral margins of the third and fourth metacarpal bones. After dissection of the extensor retinaculum, it was observed that the proximal insertion of the EDBM occurred deep to this structure, over the dorsal radiocarpal ligament, in the distal portion of the radius (Fig. 1B); its distal insertion was located on the medial margin of the extensor expansion of the middle finger. (Fig. 1C). Morphometric analysis revealed that the EDBM muscle belly measured 47.88 mm in length and 6.70 mm at its widest point, while the tendon measured 46.87 mm in length and 2 mm in width.

## Discussion

This study describes a type III EDBM – the presence of the EIP and its distal attachment to the extensor hood of the middle finger are consistent with the type III variant postulated by Ogura et al. [4]. In addition to the fact that EDBM detection in cadavers is infrequent, with a pooled prevalence of 1.9% in a sample of 9.686 hands, insertion into the middle finger by a distinct tendon had a pooled prevalence of 0.19%, being the least representative variant among the types documented in the literature [2]. Functionally, it is possible to assume that the present EDBM has an auxiliary function in extending the finger and can be considered a proper extensor muscle of the middle finger [4]. Nevertheless, since the tendon attaches to a single region of the extensor hood, it can be assumed that this muscle performs the abduction of the middle finger as demonstrated by Vaghela et al. [8].

Not all EDBM findings fit the classification of Ogura et al. Atypical cases have been reported in the Brazilian population, including insertions into the fascia of the dorsal interosseous muscle, suggesting that its variability may exceed the three classical types [2]. Proximal origins also vary and may include the radius, capitate, hamate, metacarpal bases, or an extension of the tendon of the extensor carpi radialis brevis. Although the present case shows a single muscle belly, double-bellied variants acting on the third finger have also been described [9].

The EDBM may have originated from the deep portion of the precursor extensor muscle mass of the forearm, which differentiates into radial, superficial, and deep segments during human embryonic development. While the superficial portion exhibits evolutionary stability, the deep portion is highly variable and gives rise to the abductor pollicis longus, extensor pollicis brevis, extensor pollicis longus, and the EIP. Owing to this developmental instability, the EDBM is frequently regarded as a variant of the EIP or as an atavistic muscle resulting from a failure of proximal migration of the ulnocarpal elements within the extensor muscle mass [3]. Regarding its blood supply and innervation, the EDBM shares with the EIP, which are, respectively, derived from the posterior interosseous artery and the posterior interosseous nerve, as confirmed in the specimen described in this study [6].

Its clinical significance is extremely important for medicine. The occurrence of EDBM is usually silent. However, when symptomatic, chronic pain in the back of the wrist is the main symptom in patients. In 1999, the term “fourth compartment syndrome” was applied by Hayashi et al. to refer to chronic pain generated by structures deep to the extensor retinaculum. Among the five possible etiologies of this symptom, EDBM was identified as one among them, along with carpal bone deformities, tenosynovitis, abnormalities in the extensor muscle of the index finger, and the presence of a ganglion [5]. The use of the term ‘ganglion’ alone generates imprecision, since conceptually a ganglion is a cluster of neurons present in the peripheral part of the nervous system [10]. However, the term ‘ganglion’ may have been used to refer to a single lymph node or a group of lymph nodes in the region. Regardless of the situation, given the topography of EDBM occurrence, there is no support in the literature to claim that the muscle in question can be confused with a cluster of neuronal cell bodies or a lymph node.

When pain during wrist extension is associated with work activities, EDBM tends to become a medical case. A study including 3,404 adult patients revealed that 19 of the 38 identified cases of EDBM presented symptoms severe enough to warrant surgical procedures in the region [11]. Surgically, there are two treatment solutions: retinacular release and muscle belly excision. Patel et al. suggested that the first therapeutic approach should be retinacular release, especially when EIP is compensated by EDBM, provided that the latter is normal in electrophysiological and histopathological exams, or if it can be effective in restoring the function of the extensor pollicis longus. When retinacular release is ineffective, or when the patient prefers it for aesthetic reasons, muscle belly excision is recommended as definitive treatment [7].

Recently, Alkabbaa and Abdullah (2026) described a 25-year-old man with EBDM bilaterally, whose main symptom was pain during hand manipulations, especially during exercises, affecting his daily activities. According to the patient, surgeons decided to excise the muscle, offering the patient relief of the symptoms with an uneventful postoperative course [12]. For a better surgery plan, the ultrasound and the magnetic resonance might be the radiologic procedures applied to measure the tendon, the belly, and to study the adjacent structures before the resection [13].

There is still no exclusive clinical treatment for symptomatic carriers of this variant that is widely disseminated and approved by the medical community. Botulinum toxin has been used as an alternative to surgery, being applied directly to the muscle, with a significant relief response in wrist pain and muscle spasm episodes in a patient with EDBM [14]. However, there are still no randomized clinical trials in the literature regarding this treatment, and further studies are needed so that surgery can cease to be the only way to successfully treat those with EDBM whose functions are limited by the symptoms caused by this anatomical variation.

## Conclusion

EDBM is a significant variation, both anatomically and clinically. The low occurrence rate of this variation, especially the type III variant described here, highlights the importance of knowledge and accuracy in its identification by healthcare professionals. Understanding the anatomical characteristics and clinical implications of EDBM is essential to improve existing treatment models, particularly in cases where the presence of the muscle causes discomfort and/or functional limitations in patients. Currently available therapeutic approaches offer healthcare professionals the possibility of individualizing management according to the needs and desires of each patient, but unfortunately, they still depend on surgery for resolution. Therefore, continuous study and expansion of knowledge about EDBM are essential to ensure more effective and less invasive treatments, as well as to promote quality of life for symptomatic individuals with this rare anatomical variation.

## Data Availability

No datasets were generated or analysed during the current study.
